# A Real-World Multicentre Retrospective Study of Low-Dose Apatinib for Human Epidermal Growth Factor Receptor 2-Negative Metastatic Breast Cancer

**DOI:** 10.3390/cancers14174084

**Published:** 2022-08-23

**Authors:** Tianyu Zeng, Chunxiao Sun, Yan Liang, Fan Yang, Xueqi Yan, Shengnan Bao, Yucheng Zhang, Xiang Huang, Ziyi Fu, Wei Li, Yongmei Yin

**Affiliations:** Department of Oncology, The First Affiliated Hospital of Nanjing Medical University, 300 Guangzhou Road, Nanjing 210029, China

**Keywords:** apatinib, HER2-negative, metastatic breast cancer, efficacy, breast cancer susceptibility gene

## Abstract

**Simple Summary:**

There are limited treatments for patients with HER2-negative breast cancer, especially for patients with metastatic breast cancer. This study aimed to observe the efficacy and safety of low-dose apatinib combined with chemotherapy in real-world clinical diagnosis and treatment of HER2-negative metastatic breast cancer. A total of 128 patients were treated with 250 mg apatinib, and we observed the median progression-free survival (PFS) and overall survival (OS) were 4.7 months and 15.3 months, respectively. This observation also indicated that the breast cancer susceptibility gene (BRCA) mutation predicted a better response to apatinib and that combination of immunotherapy or paclitaxel-platinum regimens might be an optimal treatment option. Importantly, a lower dose of apatinib showed mild side effects that improved the patients’ life quality.

**Abstract:**

Treatment options for human epidermal growth factor receptor (HER2)-negative breast cancer patients are limited in comparison to the HER2-positive patients, particularly for metastatic breast cancer patients. Apatinib is a small-molecule tyrosine kinase inhibitor that targets the vascular endothelial growth factor receptor 2 (VEGFR-2). Here, we reported the apatinib-based therapy data in HER2-negative metastatic breast cancer. Apatinib was taken at a dose of 250 mg orally once per day and combined with standard chemotherapy regimens. The PFS and OS of 128 patients were 4.7 months and 15.3 months, respectively. The objective response rate (ORR) and the disease control rate (DCR) were 22.7% and 80.5%, respectively. Patients with breast cancer susceptibility gene (BRCA) mutations were found to have a longer PFS and OS. Moreover, combination immunotherapy or paclitaxel-platinum regimens shared an improved response to other regimens. Most of the adverse effects (hypertension, anaemia, and hand-foot syndrome) were grade 1 to 2. Metastatic breast cancer patients could benefit from apatinib therapy at a low dosage, and the adverse effects are mild in real-world clinical practice. Furthermore, BRCA may be a putative biomarker for apatinib in HER2-negative breast cancer. Immunotherapy or paclitaxel-platinum regimens may be recommended to combine with apatinib therapy.

## 1. Introduction

Breast cancer is the most frequently diagnosed cancer worldwide, with the highest malignancy frequency and representing the second leading cancer-related cause of death among females globally. There are almost 1,350,000 new diagnosed cases of breast cancer and approximately 500,000 deaths resulting from it around the world each year, of which Chinese cases accounted for 12.2% and 9.6%, respectively [1]. Approximately 4–6% of patients were in the metastatic stage at their initial diagnosis, and 30–40% of early breast cancer patients will eventually endure cancer relapse after adjuvant treatment. The prognosis of metastatic breast cancer patients with two or even more lines of treatment is poor, and their median survival time is only approximately one year [2,3,4,5,6,7,8]. There is an increasing need to improve the OS of patients with metastatic or recurrent metastatic breast cancer. Novel strategies are expected to ensure continued improvements. Tumor infiltration and metastasis are closely related to the vascular endothelial growth factor’s overexpression (VEGF) [9]. Research has shown that the signal transduction pathway that is mediated by the VEGF and its corresponding receptor makes an important impact on regulating breast cancer angiogenesis [10]. Apatinib is a novel small-molecule receptor tyrosine kinase inhibitor that inhibits the VEGFR-2 tyrosine kinase’s activity specifically, blocks the VEGF binding to its receptor signalling, and thus potently inhibits tumor angiogenesis. Prior studies have shown that apatinib can significantly improve the PFS and OS in refractory metastatic gastric cancer patients [11,12,13,14].

In recent years, apatinib was used for treating breast cancer and showed a good clinical benefit in both triple negative and non-triple-negative metastatic breast cancer [15,16,17,18,19,20,21]. However, whether in clinical trials and real-world studies, high doses of 500 mg or 425 mg are often chosen for treatment, resulting in an inability to tolerate grade III hypertension and proteinuria. This has often forced patients to stop tyrosine kinase inhibitor (TKI) treatment, which has been a problem for clinicians and patients [16,17,20,21]. At the same time, recent studies have shown that apatinib therapy with a low dose could improve the efficacy of anti-tumor therapy by normalizing blood vessel with tolerable adverse events. However, data from real-word studies looking at low-dose apatinib in patients with metastatic breast cancer are still missing. Therefore, we tried to add a small dose of apatinib (250 mg once daily orally) to the standard chemotherapy regimen, which avoided the withdrawal of TKI treatment due to the severe side effects. Fortunately, a good clinical effect was observed.

This study retrospectively analyzed the clinical data of patients who have metastatic breast cancer and were treated with low dose apatinib and evaluated its clinical efficacy and adverse reactions in the breast cancer field, which might provide a new method for clinical treatment.

## 2. Materials and Methods

### 2.1. Clinical Data

This research comprised 128 patients with metastatic breast cancer treated in Jiangsu Province Hospital (Nanjing, China) and Anhui Province Hospital (Anhui, China) from August 2016 to May 2021. Female patients with a measurable lesion by RECIST version 1.1 with pathological confirmation of HER2 negative breast cancer with local or distant organ metastasis were enrolled. Other inclusion conditions were having no uncontrollable underlying disease, 0 to 2 ECOG performance status, and more than three months of expected survival time. Patients with uncontrollable hypertension were excluded. All patients were assessed for routine blood, liver, kidney, and cardiac functions before administration. The Nanjing Medical University’s First Affiliated Hospital’s Ethics Committee approved this research following the Declaration of Helsinki. Written consent was taken from all participants.

### 2.2. Treatment

Apatinib was taken at a dose of 250 mg orally once per day. Blood and urine routines, blood pressure, and liver and kidney functions were examined throughout the treatment. The treatment efficacy was determined using CT or MRI following every two treatment cycles (q 8 weeks) until disease progression. Patients were treated with combined chemotherapy, such as paclitaxel liposome (175 mg/m^2^, q 21 days, *n* = 34), paclitaxel combined with cisplatin (paclitaxel liposome: 150 mg/m^2^,q 21 days; cisplatinum: 75 mg/m^2^, days 1, 2 of 21 days, *n* = 15), gemcitabine (1000 mg/m^2^, d1, 8 of 21 days, *n* = 24), capecitabine (1000 mg/m^2^, bid, d1–14 of 21 days, *n* = 19), pemetrexed (500 mg/m^2^, q 21 days, *n* = 4), vinorelbine (25 mg/m^2^, d1, 8 of 21 days, *n* = 21). In addition, four patients received camrelizumab (200 mg, q 14 days) intravenous injection in combination with apatinib.

### 2.3. Efficacy Assessments

Efficacy was assessed according to RESIST 1.1 [22], including complete remission (CR), partial remission (PR), stable disease (SD), and progression disease (PR), respectively. Progression-free survival (PFS) refers to the period between the therapy initiation and the tumor growth or death; overall survival (OS) refers to the period between drug administration and death. Objective response rate (ORR) refers to the CR+PR cases number/total cases number * 100%. Disease control rate (DCR) refers to the CR+PR+SD cases number as a percentage o total cases number. Clinical benefit rate (CBR) refers to the complete remission, partial remission, and stable disease summation as a percentage of total cases number, with a time greater than or equal to 24 weeks. The adverse effects were determined following the National Cancer Institute Common Toxicity Criteria for Adverse Events version 4.0 (NCI-CTCAEV 4.03).

### 2.4. Statistical Analyses

The statistical analysis was carried out using the SPSS22.0 (Chicago, IL, USA). The measurement data analysis had been carried out with a *t*-test. The log-rank test compared the survival analysis. The prognostic factors analysis used a multivariate cox regression model. A *p* < 0.05 was regarded as statistically significant.

## 3. Results

### 3.1. Baseline Patient Characteristics

The 128 patients’ median age was 51 (24–84 years). 14 patients were treated with apatinib as a first-line treatment, while the numbers of patients with second-line therapy and third-line therapy were 42 and 32, respectively. The remaining patients (*n* = 40) failed their multi-line therapy and had heavy treatment. The basic characteristics are depicted in Table 1. 49.2% (63/128) of patients was TNBC and 89.8% (115/128) of patients had visceral metastases. The lung, liver, and bone are the most common metastasis sites. Importantly, more than half of patients have at least two metastatic lesions.

### 3.2. Efficacy Outcome

Consequently, all 128 patients obtained at least 2 cycles of treatment and were therefore evaluable. All 128 patients were enrolled in the analysis, and the median PFS and the OS were 4.7 months (95% CI: 3.9–5.5 months) and 15.3 months (95% CI: 13.9–16.7 months), respectively.

**Figure 1:**
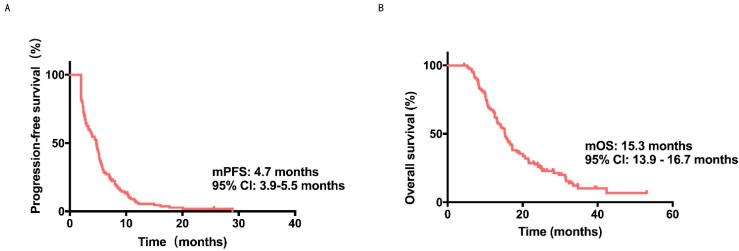
The PFS and OS’s KaplanMeier curve of metastatic HER2negative breast cancer patients treated with apatinib. (**A**) PFS KaplanMeier curve, which indicated a 4.7 months median PFS (95% CI = 3.9–5.5). (**B**) OS Kaplan-Meier curve, which indicated a 15.3 months median OS (95% CI = 13.9–16.7).

Surprisingly, 5 patients endured no tumor progression, and 25 patients stayed alive by the follow-up end (Figure 1A,B). There were no patients with CR, 29 patients with PR, 74 patients with SD, and 25 patients with PD (Table S1). The ORR, DCR, and CBR were 22.7%, 80.5%, and 38.3%, respectively. 

No considerable differences in PFS and OS between TNBC and HR (*p* = 0.109 and *p* = 0.061, respectively, according to the inter-group analysis (Figure S1A,B, Table 2). According to the number of treatment lines, the patients were divided into two categories, namely <3-line treatment and multi-line treatment (≥3 lines). The <3-line and the multi-line treatment groups’ median PFS were 5.1 and 3.5 months, respectively (*p* = 0.034, Figure S1C, Table 2). Unfortunately, no considerable differences were found in OS between these patients [17.1 months vs. 14.7 months, *p* = 0.239; Figure S1D, Table 2].

**Figure 2:**
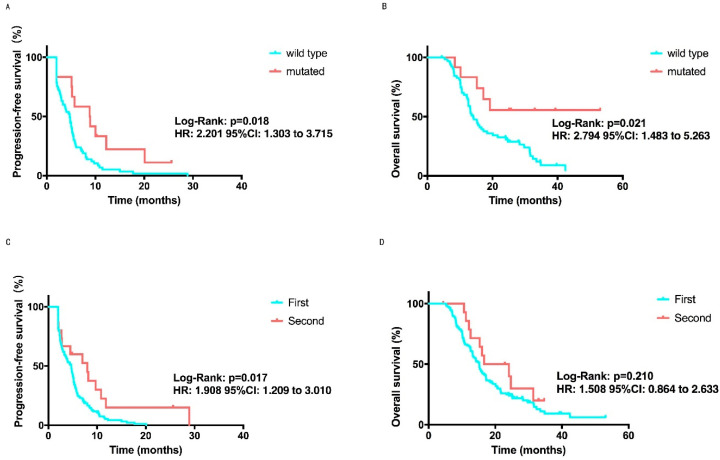
PFS and OS Kaplan-Meier curves in subgroup analysis. (**A**) PFS Kaplan-Meier curve comparing mutated BRCA patients, with a 8.9 months median PFS, and those who were unmutated, with a 4.7 months median PFS. *p* = 0.018 was considered a statistically significant. (**B**) OS Kaplan--Meier curve comparing mutated BRCA patients with a not reached median OS and those who were unmutated, with a 14.1 months median OS. *p* = 0.021 was considered a statistically significant. (**C**) PFS Kaplan-Meier curve comparing patients treated with apatinib for the first time, with a 4.7 months median PFS, and those who were second time, with 8.0 months median PFS. *p* = 0.017 was considered a statistically significant (**D**) OS Kaplan-Meier curve comparing patients treated with apatinib for the first time, with a 15.2 months median OS, and those who were second time, with a 16.6 months median OS. No significant difference was found (*p* = 0.210).

Seventy-seven patients did genetic testing. The data analysis of these patients revealed that the median PFS of patients with BRCA mutations reached 8.9 months, while the PFS of the unmutated group was only 4.7 months (*p* = 0.018, Figure 2A, Table 2). The same tendency was observed in OS analysis (*p* = 0.021, Figure 2B, Table 2). Among all of the patients, 15 patients had continued treatment with the apatinib and another chemotherapy agent combination after PD for the first time. No considerable differences were observed in OS among patients treated with (16.6 m, 95%CI: 6.2–27.0 m) or without apatinib (15.2 m, 95% CI: 13.5–17.0 m, *p* = 0.210, Figure 2D) according to an inter-group analysis, but different in PFS (8.0 vs. 4.7 m, 95% CI: 2.0–14.0 months, 95% CI: 3.9–5.5 months, *p* = 0.0.017, Figure 2C, Table 2).

When exploring the relations between efficacy and chemotherapy regimens, the eight groups showed significantly different PFS, and apatinib combined with immunotherapy achieved the highest PFS among the groups (11.8 months vs. 7.1 months vs. 5.7 months vs. 4.6 months vs. 4.4 months vs. 3.2 months vs. 2.8 months vs. 2.5 months, *p* < 0.001, for group immunotherapy, paclitaxel and platinum, paclitaxel, pemetrexed, epirubicin, vinorelbine, gemcitabine and capecitabine, respectively. Supplementary Figure S2A, Table 2). Similarly, the same trend toward OS in the immunotherapy group were also observed (*p* = 0.001, Supplementary Figure S2B, Table 2).

**Figure 3:**
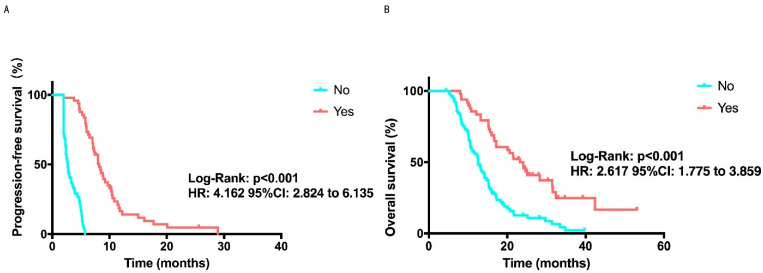
PFS and OS’s Kaplan-Meier curves in clinical benefit analysis. (**A**) PFS Kaplan-Meier curve comparing patients that received a clinical benefit, with a 8.2 months median PFS, and those who did not receive a clinical benefit, with a 2.7 months median PFS. *p* < 0.001 was considered a statistically significant (**B**) OS Kaplan-Meier curve comparing patients that received a clinical benefit, with a 24.1 months median OS, and those who did not receive a clinical benefit, with a 12.6 months median OS. *p* < 0.001 was considered a statistically significant.

To seek predictive biomarkers of apatinib, the other characteristics (Table 2) and efficacy’s correlations were assessed. Patients who didn’t receive radiotherapy (*p* = 0.003, Figure S3A,B), without bone metastasis (*p* = 0.014, Supplementary Figure S4A,B), with elevated blood pressure (*p* = 0.012, Supplementary Figure S5A,B), with the hand-foot syndrome (*p* = 0.023, Supplementary Figure S5C,D) and achieve clinical benefit (*p* < 0.001), were significantly correlated with prolonged PFS. At the same time, without axillary lymph nodes (*p* = 0.024, Supplementary Figure S4C,D) and achieved clinical benefit (*p* < 0.001, Figure 3A,B) were the predictor of longer OS. Meanwhile, no considerable difference was found in PFS or OS between these patients with Ki67 (Figure S3C,D), brain metastasis (Supplementary Figure S2E,F), chest wall metastasis (Supplementary Figure S4G,H), liver metastasis (Supplementary Figure S4I,J), lung metastasis (Supplementary Figure S4K,L), proteinuria (Supplementary Figure S5E,F), micropapillary (Supplementary Figure S6A,B) and vascular cancer embolus (Supplementary Figure S6C,D) with those who did not.

A multivariate model with all of the variables (Table S2) was also attempted. In Cox multivariate analysis, the BRCA mutation and clinical benefit were linked to a considerably longer PFS (*p* = 0.016, *p* = 0.001, respectively). Additionally, a significantly prolonged OS was observed in HR+ patients (*p* = 0.003), Ki67 ≤ 15% patients (*p* = 0.001), patients without axillary lymph nodes metastasis (*p* = 0.021) and patients with <3 lines of treatment receiving apatinib (*p* = 0.049).

### 3.3. Safety

The AEs that most frequently occurred were hypertension, anorexia, hand-foot syndrome, leukopenia, and anemia. Toxicities were mainly well-tolerated, and all of them could be regulated by symptomatic treatments. Notably, no serious adverse events occurred, and none of them resulted in treatment-related deaths (Table 3).

## 4. Discussion

Cancer metastases are a challenge for cancer therapy due to their organ specificity and pathophysiological complexity. Recent reports on in vitro 3D culture [23,24,25] have significantly improve our understanding of cancer metastasis and helped to guide the development of more effective treatments. However, the existing therapy for metastatic breast cancer patients are still not enough. Especially, HER2-negative breast cancer patients had lost the chance of HER2-targeted therapy and may fail in more than third-line treatments. Therefore, HER2-negative breast cancer patients need extra treatment options that are generally made based according to the doctor’s experience and the patient’s condition. Apatinib is an oral small-molecule VEGFR2 inhibitor. The anti-angiogenic drugs’ mechanism involves normalizing the abnormal tumor blood vessels and then promoting the influx of more chemotherapy or endocrine therapy drugs into the tumor, thus improving the efficacy of the above drugs (which is a synergistic treatment) [8,26]. At the same time, preclinical data have shown that apatinib could enhance the paclitaxel and cisplatin’s anti-tumor effect [27,28]. Additionally, apatinib can inhibit the chemotherapeutic drugs’ efflux [29] and reverse the tumor cells’ multidrug resistance [30]. We hypothesize that the combination of chemotherapy and apatinib may be a therapeutic option for metastatic HER2-negative breast cancer patients.

Most patients (*n* = 72, 56.3%) in our study were heavily treated (≥3 lines) and it was difficult to formulate a standard therapeutic schedule. In general, the non-response or the resistance to cytotoxic agents results in decreased efficiency of future chemotherapy lines, with 10 and 20% ORR range [3,31]. As shown in Table S3, Hu et al. [6,7] performed two prospective, multicenter, phase II trials to estimate the efficacy of apatinib as a single treatment in patients with pretreated metastatic TNBC or non-TNBC. Following their results, among 56 patients with TNBC [7] qualify for analysis, the median PFS and OS were 3.3 months and 10.6 months, respectively. Among 38 patients with advanced non-TNBC [6], apatinib acquired a median PFS and OS of 4.0 months and 10.3 months, respectively. In addition, the retrospective analysis results revealed that metastatic breast cancer patients had comparable results compared with other trials [15,16,17,18,20,21,32,33] after treatment with apatinib. The median PFS and the OS were 4.7 and 15.4 months, respectively, and the longer period than patients in prior chemotherapy alone studies [3,31,34,35,36,37,38,39,40] suggested that apatinib may be used to treat individuals who have developed multidrug resistance to traditional chemotherapy medicines, while the ORR and the DCR were 22.7%, and 80.5%, respectively. The HeCOG trial [35] share a median PFS and OS of 3.7 and 15.2 months as response to the cabazitaxel at second line therapy. Moreover, clinical trials NCT03044730 [36], EMBRACE [3], Study 301 [37], BEACON [38] and Study 304 [39] have reported the median PFS from 2.2 to 4.2 months and OS from 10.6–15.9 months. By comparing these results, our results are convincing, especially since our trial included a larger proportion of triple negative breast cancer patients, and our treatment lines were further behind since more patients were treated with multiple lines. At the same time, this real-world study is more complicated and closer to clinical reality than a prospective study.

In clinical settings, the common daily doses of apatinib are either 850 mg or 500 mg, but several adverse reactions frequently emerge from this treatment. Patients often have symptomatic relief after symptomatic treatment, but they have to stop taking the drug due to uncontrollable hypertension and proteinuria, which weakens the treatment effect. A Phase IIb clinical trial found that a 1/3 reduction of apatinib could reduce the adverse reactions’ incidence and degree, and the drug efficacy was not affected [7]. It has been established that the 500 mg apatinib has tolerable toxicity, so patients who received multiline rescue chemotherapy at a later stage were treated at a dose of 250 mg orally once per day in this research. The most common adverse reactions were mostly mild (Grade 1~2), such as hypertension, anorexia, hand-foot syndrome, leukopenia, and fatigue, and all of these symptoms had been improved after symptomatic treatment. No intolerable side effects were found, and no treatment-related death occurred. These findings revealed that low dose apatinib reduced patients’ side effects and economic burden and achieved good curative effects, providing a new method for metastatic breast cancer treatment.

Despite the fact that no statistical difference was discovered in either PFS or OS between various molecular types, and patients substantially vary in responsive to therapy considering the heterogeneous nature of breast cancer, predictive biomarkers are urgently needed for recognizing the patients sensitive to apatinib and avoiding the exposure to the unnecessary toxic agents rather than blind over-medication. Previous studies have shown that pVEGFR in tumor tissue, sVEGFR2 in serum, and treatment-related side effects after treatment are related to the prognosis of patients [41,42]. However, it has also been reported that only the expression of pVEGFR2 in tumor tissues can predict the clinical benefit and progression-free survival of breast cancer patients receiving apatinib and VEGF or sVEGFR2 in serum cannot accurately predict the prognosis of patients [7]. Our results found that patients with BRCA mutations benefited more from apatinib with a 8.9 months median PFS (*p* = 0.018), but they did not reach median OS (*p* = 0.021). This finding has not been reported in other studies. Basic studies have reported that VEGF3 inhibitors can elevate the chemical sensitization of ovarian cancer stem cells by downregulating BRCA1/2 [43], but the specific mechanism in breast cancer still needs further exploration with basic experiments. We also found that patients without radiation before apatinib therapy benefit more than those who received radiation (*p* = 0.003). The increased blood vessel wall fibrosis resulting from radiotherapy can weaken the apatinib effect. In addition, patients who received fewer treatment lines (*p* = 0.034), without bone metastasis (*p* = 0.014), and with clinical benefit (*p* < 0.001) shared prolonged PFS as we envisioned. According to our results, patients with axillary lymph nodes metastasis (*p* = 0.024) with longer OS demonstrating potential antitumor activity of apatinib due to lymph angiogenesis may be related to the VEGFC/VEGFR3 pathway [44]. Additionally, prior exposure to apatinib was an independent predictor of longer PFS (*p* = 0.017) but not OS (*p* = 0.210), suggesting that prior anti-angiogenic therapy is not contraindicated with apatinib. Nevertheless, due to the lack of prospective controlled trials, we do not know whether each patient should start apatinib combined with other drugs as a next-line treatment rather than suspend anti-angiogenic treatment. Moreover, patients with hypertension (*p* = 0.012) and hand-foot syndrome (*p* = 0.023) after receiving apatinb treatment benefit more than those without symptoms, which is consistent with previous studies [15,16,17].

In addition, this real-world study showed that the combined treatment schedule might affect PFS and OS. Among them, apatinib combined with immunotherapy achieved the highest PFS (11.8 months) and the not reach OS, which is in keeping with the study of the camrelizumab and apatinib combination in metastatic triple-negative breast cancer [18]. Subsequently, paclitaxel and platinum obtained the 7.1 months PFS and the 25.1 months OS, and paclitaxel gained the 5.7 months PFS and the 20.6 months OS. Nevertheless, some patients refuse accepting immunotherapy due to the high economic evaluation. Based on our research findings, apatinib combined with paclitaxel and platinum or paclitaxel may be regarded a salvage therapy option for those who can’t receive immunotherapy. Moreover, our multivariate COX analysis showed that BRCA was the predictive biomarker of PFS and molecular type, Ki67, axillary lymph nodes, lines of treatment, and the chemotherapy regimen were used to predict patient survival outcomes. Certainly, all of these findings should be verified in further study.

This study is an observational study which focuses on the evaluation of low dose apatinib treatment in the real world. Limitations such as the small sample size, lack of randomized controlled studies, and confounding factors may inevitably lead to bias Further research is necessary. However, these research results demonstrated that apatinib-based combined therapy could be effective for heavily treated metastatic HER2-negative breast cancer. The mutated BRCA can be a predictor for the apatinib efficiency. Considering the manageable toxicity and considerable therapeutic effect, apatinib presents a new alternative treatment for metastatic HER2-negative breast cancer patients.

## 5. Conclusions

Our study indicated that patients with advanced HER2-negative breast cancer will benefit from low dose (250 mg) apatinib treatment. This observation indicated that the BRCA mutation predicted a better response to apatinib and apatinib combined withimmunotherapy or paclitaxel-platinum regimens may be an optimal treatment option. Importantly, a lower dose of apatinib showed mild side effects that improved the quality of life of the patients.

## Figures and Tables

**Table 1:** Baseline data for patients with metastatic breast cancer.

Characteristics	No.	%
ECOG performance status		
0–1	62	48.4
2	66	51.6
ER		
positive	65	50.8
negative	63	49.2
PR		
positive	52	40.6
negative	76	59.4
Number of prior systemic therapy lines		
1	14	10.9
2	42	32.8
3	32	25.0
>3	40	31.3
Metastatic sites		
bone	59	46.1
liver	56	43.8
lung	69	53.9
chest wall	24	18.8
brain	11	8.6
Ki67		
≤15	16	12.5
>15	94	73.4
Unknown	18	14.1
Axillary lymph node		
positive	79	61.7
negative	49	38.3
Radiotherapy		
yes	72	56.3
no	56	43.7
Age		
≤50	61	47.7
>50	67	52.3
Type of metastatic sites		
Visceral	115	89.8
Only non-visceral	13	10.2

**Table 2:** Subgroup analysis comparing median PFS and OS among patients with different characteristics.

Characteristic	Progression-Free Survival		Overall Survival	
	Median PFS, Months (95% CI)	*p*-Value	Median OS, Months (95% CI)	*p*-Value
Age, years		0.442		0.328
≤50	4.5 (3.0–6.0)		14.2 (11.4–17.0)	
>50	4.9 (4.2–5.6)		16.0 (14.4–17.6)	
Molecular type		0.109		0.062
HR+	4.0 (2.6–5.4)		17.2 (15.0–19.4)	
TNBC	5.1 (4.6–5.6)		10.7 (8.5–12.9)	
Ki67		0.425		0.133
≤15%	5.0 (4.1–5.9)		15.7 (13.7–17.7)	
>15%	4.5 (3.1–5.9)		14.2 (11.7–16.7)	
Micropapillary		0.390		0.514
No	4.8 (4.0–5.6)		15.3 (13.9–16.7)	
Yes	3.9 (2.4–5.4)		NR	
Vascular cancer embolus		0.890		0.173
No	4.7 (3.8–5.6)		15.2 (13.8–16.6)	
Yes	4.7 (2.8–6.6)		17.2 (9.7–24.7)	
Axillary lymph nodes		0.697		0.024
No	3.9 (2.3–5.5)		15.2 (12.3–18.1)	
Yes	4.8 (4.2–5.4)		15.3 (13.3–17.3)	
Radiotherapy		0.003		0.521
No	5.2 (4.1–6.3)		16.6 (13.0–20.2)	
Yes	3.9 (2.3–5.5)		15.2 (13.7–16.7)	
BRCA		0.018		0.021
Mutated	8.9 (3.5–14.3)		NR	
Wild type	4.7 (3.7–5.7)		14.1 (12.1–16.1)	
Lines of treatment		0.034		0.213
<3	5.1 (4.8–5.4)		17.1 (14.6–19.6)	
≥3	3.5 (2.6–4.4)		14.7 (12.7–16.7)	
Liver metastasis		0.125		0.869
No	5.1 (4.4–5.8)		14.1 (12.5–15.7)	
Yes	3.9 (2.4–5.4)		17.2 (14.9–19.5)	
Lung metastasis		0.456		0.156
No	3.5 (2.0–5.0)		14.1 (11.9–16.3)	
Yes	5.0 (4.5–5.5)		16.6 (14.1–19.1)	
Brain metastasis		0.834		0.673
No	4.7 (3.9–5.5)		15.3 (13.7–16.9)	
Yes	5.3 (2.3–8.3)		15.2 (10.8–19.6)	
Chest wall metastasis		0.769		0.920
No	4.5 (3.6–5.4)		15.2 (13.5–16.9)	
Yes	5.1 (3.9–6.3)		15.7 (12.5–18.9)	
Bone metastasis		0.014		0.107
No	5.2 (4.8–5.6)		17.1 (14.8–19.4)	
Yes	3.8 (2.3–5.3)		14.7 (12.7–16.7)	
Hypertension		0.012		0.232
No	3.8 (1.8–5.8)		14.2 (12.1–16.3)	
Yes	4.8 (4.1–5.6)		16.2 (14.1–18.3)	
Hand-foot syndrome		0.023		0.226
No	3.9 (2.6–5.2)		14.7 (12.3–17.1)	
Yes	5.3 (4.4–6.2)		16.2 (14.4–18.0)	
Proteinuria		0.500		0.592
No	4.7 (3.9–5.5)		15.3 (14.0–16.6)	
Yes	4.0 (1.4–6.6)		13.7 (9.7–16.9)	
Apatinb order		0.017		0.210
First	4.7 (3.9–5.5)		15.2 (13.5–17.0)	
Second	8.0 (2.0–14.0)		16.6 (6.2–27.0)	
Chemotherapy regimen		<0.001		0.001
Immunotherapy	11.8 (2.0–21.6)		NR	
Paclitaxel and platinum	7.1 (5.4–8.8)		25.1 (8.5–41.7)	
Paclitaxel	5.7 (4.3–7.1)		20.6 (17.3–23.9)	
Pemetrexed	3.3 (1.2–5.4)		8.1 (2.0–14.6)	
Epirubicin	4.0 (1.8–6.2)		16.6 (8.6–24.6)	
Vinorelbine	3.0 (2.2–3.8)		12.6 (6.3–18.9)	
Gemcitabine	2.7 (1.4–4.0)		12.2 (10.2–14.2)	
Capecitabine	2.5 (2.0–3.0)		13.2 (9.8–16.6)	
Clinical benefit		<0.001		<0.001
No	2.7 (2.4–3.0)		12.6 (11.0–14.2)	
Yes	8.2 (7.0–9.4)		24.1 (19.6–28.6)	

**Table 3:** Adverse events.

AE	Grade 1	Grade 2	Grade 3	Grade 4
Hypertension	42 (32.8)	23 (18.0)	13 (10.2)	0
Anemia	19 (14.8)	11 (8.6)	3 (2.3)	0
Proteinuria	16(12.5)	6 (4.7)	1 (0.8)	0
Hand-foot syndrome	18 (14.1)	30 (23.4)	7 (5.5)	0
Thrombocytopenia	18 (14.1)	9 (7.0)	3 (2.3)	0
Leukopenia	27 (21.1)	13 (10.2)	5 (3.9)	1 (0.8)
Hepatotoxicity	17 (13.3)	11 (8.6)	1 (0.8)	0
Fatigue	18 (14.1)	27 (21.1)	3 (2.3)	0
Anorexia	28 (21.)	31 (24.2)	10 (7.8)	0

## Data Availability

The data analyzed during the research are included in this study or are available on reasonable request.

## Supplementary Materials

The following supporting information can be downloaded at: www.mdpi.com/article/10.3390/cancers14174084/s1, Table S1: Best clinical response; Table S2: Multivariate COX analyses of factors associated with PFS and OS; Table S3: Previous studies on treatment for metastatic breast cancer; Figure S1: Kaplan-Meier curves of PFS and OS in subgroup analysis; Figure S2: Kaplan-Meier curves of PFS and OS in combination regimens; Figure S3: Kaplan-Meier curves of PFS and OS in subgroup analysis; Figure S4: Kaplan-Meier curves of PFS and OS in metastasis location; Figure S5: Association between PFS or OS and side effects after treatment; Figure S6: Kaplan-Meier curves of PFS and OS in pathology analysis.
