# Supplementary material for: A Real-World Multicentre Retrospective Study of Low-Dose Apatinib for Human Epidermal Growth Factor Receptor 2-Negative Metastatic Breast Cancer
Source: Cancers (Basel). 2022 Aug 23;14(17):4084. doi: 10.3390/cancers14174084 (PMC9454649; doi:10.3390/cancers14174084)
Supplement: Supplementary file 1 [file cancers-14-04084-s001.zip › cancers-1849997-supplementary.pdf]

Article

A Real-World Multicentre Retrospective Study of Low-Dose Apatinib for Human Epidermal Growth Factor Receptor 2-Negative Metastatic Breast Cancer

Tianyu Zeng †, Chunxiao Sun †, Yan Liang, Fan Yang, Xueqi Yan, Shengnan Bao, Yucheng Zhang, Xiang Huang, Ziyi Fu, Wei Li * and Yongmei Yin *

Department of Oncology, The First Affiliated Hospital of Nanjing Medical University, 300 Guangzhou Road, Nanjing 210029, China

* Correspondence: real.lw@163.com (W.L.); ymyin@njmu.edu.cn (Y.Y.); Tel: +86-025-68307102 (W.L. & Y.Y.)

† These authors contributed equally to this work.

Citation: Zeng, T.; Sun, C.; Liang, Y.; Yang, F.; Yan, X.; Bao, S.; Zhang, Y.; Huang, X.; Fu, Z.; Li, W.; et al. A Real-World Multicentre Retrospective Study of Low-Dose Apatinib for Human Epidermal Growth Factor Receptor 2-Negative Metastatic Breast Cancer. *Cancers* **2022**, *14*, 4084. https://doi.org/10.3390/cancers14174084

Academic Editor: Samuel C. Mok

Received: 20 July 2022
Accepted: 20 August 2022
Published: 23 August 2022

Publisher's Note: MDPI stays neutral with regard to jurisdictional claims in published maps and institutional affiliations.

Copyright: © 2022 by the authors. Licensee MDPI, Basel, Switzerland. This article is an open access article distributed under the terms and conditions of the Creative Commons Attribution (CC BY) license (https://creativecommons.org/licenses/by/4.0/).

Table S1. Best clinical response.

Best clinical response	N	%
CR	0	0
PR	12	25.5
SD	26	55.3
PD	9	19.2

Table S2. Multivariate COX analyses of factors associated with PFS and OS.

Characteristic	Progression-free survival	Overall survival
	p-value	p-value
Age, years	0.383	0.807
Molecular type	0.434	0.003
Ki67	0.670	0.001
Micropapillary	0.418	0.260
Vascular cancer embolus	0.760	0.678
Axillary lymph nodes	0.738	0.021
Radiotherapy	0.464	0.317
BRCA	0.016	0.072
Lines of treatment	0.162	0.049
Liver metastasis	0.593	0.824
Lung metastasis	0.119	0.369
Brain metastasis	0.660	0.201
Chest wall metastasis	0.368	0.575
Bone metastasis	0.415	0.301
Hypertension	0.302	0.561
Hand-foot syndrome	0.530	0.235
Proteinuria	0.267	0.465
Apatinb order	0.326	0.740
Chemotherapy regimen	0.866	0.007
Clinical benefit	0.001	0.001

Table S3. Previous studies on treatment for metastatic breast cancer.

	HU *ET AL*	HEC OG	NCT0304 4730	EM-BRACE	STUDY 301	BEA-CON	STUD Y 304	RIB-BON-2	JACOT *ET AL*
Regimen	aptinib	caba-zitaxel	pem-broli-zumab and capecita-bine	eribulin or TPC	eribulin or cape-citabine	etiri-notecan pegol or TPC	eribu-lin or vi-norelbi n	chemo-therapy bevaci-zumab or chemo-therapy	eribulin or chem-other-apy
Enrolled patients, n	84+38	84	30	762	1102	852	530	684	10729
No. of prior chemo-therapy:	1-4	1	0-5	1-5	0-2	1-6	2-6	1	1-3
Patients with TNBC, n (%)	84 (68.9%)	26 (31%)	16 (25%)	144 (19%)	284 (25.8%)	236 (28%)	132 (24.9%)	159 (23.2%)	2092 (19.5%)
PFS (months)	TNBC: 3.3	3.7	4.0	eribu-lin:3.7	eribu-lin: 4.1	etiri-notecan	eribu-lin :2.8	chemo-therapy	eribu-lin:4.14

	Non-TNBC: 4.0			TPC: 2.2	capecit-a-bine:4.2	pegol:2.4 TPC:2.8	vi-norelbin:2.8	bevaci-zumab: 7.2 chemo-therapy: 5.1	chemo-therapy: 3.02
OS (months)	TNBC:10.6 Non-TNBC: 10.3	15.2	15.4	eribu-lin:13.1 TPC: 10.6	eribu-lin: 15.9 Cape-cita-bine:14.5	etiri-notecan pegol:12.4 TPC:10.3	eribu-lin:13.4 vi-norelbin:12.5	chemo-therapy bevaci-zumab: 18.0 chemo-therapy: 16.4	eribu-lin:11.28 chemo-therapy: 7.56

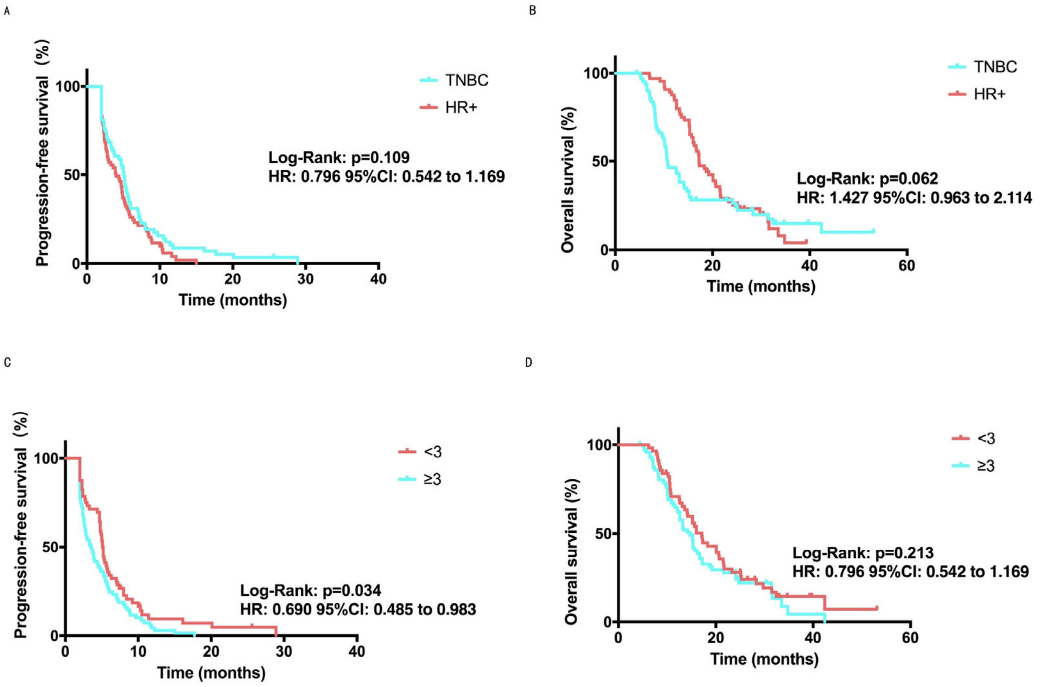

Figure S1. Kaplan-Meier curves of PFS and OS in subgroup analysis. (A) Kaplan-Meier curve of PFS comparing patients who were HR+, with a median PFS of 4.0 months, and those who were TNBC, with a median PFS of 5.1 months. No significant difference was identified between these patients (p=0.109). (B) Kaplan-Meier curve of OS comparing patients who were HR+, with a median OS of 17.2 months, and those who were TNBC, with a median OS of 10.7 months. No significant difference was identified between these patients (p=0.062). (C) Kaplan-Meier curve of PFS comparing patients who were less than 3 lines treatment, with a median PFS of 5.1 months, and those who were multi-line treatment (≥3 lines), with a median PFS of 3.5 months. A statistically significant difference was identified between these patients (p=0.034). (D) Kaplan-Meier curve of OS comparing patients who were less than 3 lines treatment, with a median OS of 17.1 months, and those who were multi-line treatment (≥3 lines), with a median PFS of 14.7 months. No significant difference was identified between these patients (p=0.213).

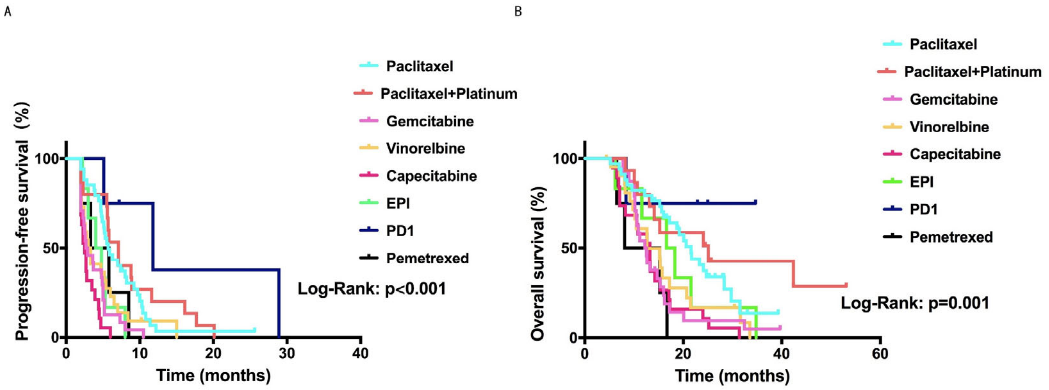

Figure S2. Kaplan-Meier curves of PFS and OS in combination regimens. (A) Kaplan-Meier curve of PFS comparing patients who combined with immunotherapy, with a median PFS of 11.8 months, who combined with paclitaxel and platinum, with a median PFS of 7.1 months, who combined with paclitaxel, with a median PFS of 5.7 months, who combined with pemetrexed, with a median PFS of 4.6 months, who combined with epirubicin, with a median PFS of 4.4 months, who combined with vinorelbine, with a median PFS of 3.2 months, who combined with gemcitabine, with a median PFS of 2.8 months, who combined with capecitabine, with a median PFS of 2.5 months. A statistically significant difference was identified between these patients ($p<0.001$). (B) Kaplan-Meier curve of OS comparing patients who combined with immunotherapy, with a not reached median OS, who combined with paclitaxel and platinum, with a median OS of 25.1 months, who combined with paclitaxel, with a median OS of 20.6 months, who combined with pemetrexed, with a median OS of 8.1 months, who combined with epirubicin, with a median OS of 16.6 months, who combined with vinorelbine, with a median OS of 12.6 months, who combined with gemcitabine, with a median OS of 12.2 months, who combined with capecitabine, with a median OS of 13.2 months. A statistically significant difference was identified between these patients ($p=0.001$).

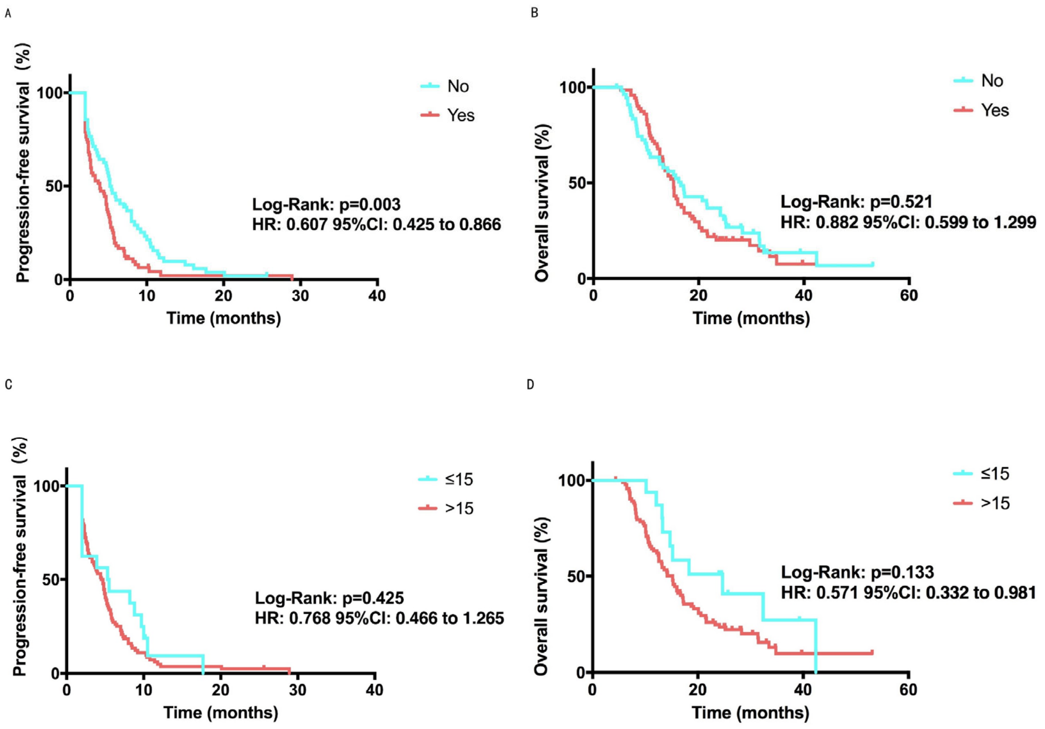

Figure S3. Kaplan-Meier curves of PFS and OS in subgroup analysis. (A) Kaplan-Meier curve of PFS comparing patients who received radiotherapy, with a median PFS of 3.9 months, and those who did not receive radiotherapy, with a median PFS of 5.2 months. A statistically significant difference was identified between these patients (p=0.003). (B) Kaplan-Meier curve of OS comparing patients who received radiotherapy, with a median OS of 15.2 months, and those who did not receive radiotherapy, with a median OS of 16.6 months. No significant difference was identified between these patients (p=0.521). (C) Kaplan-Meier curve of PFS comparing patients who with low Ki67 (≤15%), with a median PFS of 5.0 months, and those who were high Ki67 (>15%), with a median PFS of 4.5 months. No significant difference was identified between these patients (p=0.425). (D) Kaplan-Meier curve of OS comparing patients who with low Ki67 (≤15%), with a median OS of 15.7 months, and those who were high Ki67 (>15%), with a median OS of 14.2 months. No significant difference was identified between these patients (p=0.133).

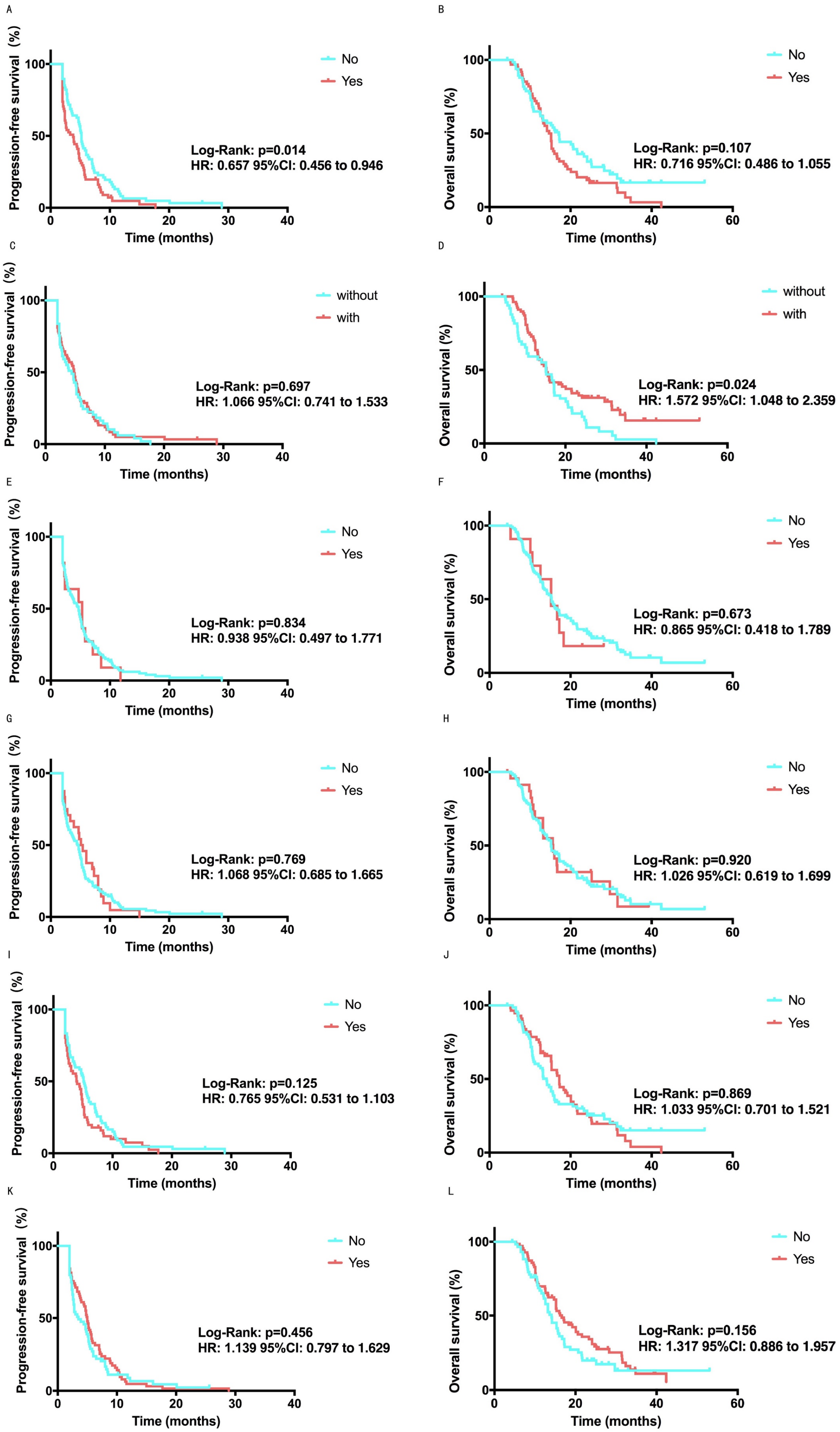

Figure S4. Kaplan-Meier curves of PFS and OS in metastasis location. (A) Kaplan-Meier curve of PFS comparing patients who with bone metastasis, with a median PFS of 3.8 months, who without

bone metastasis, with a median PFS of 5.2 months. A statistically significant difference was identified between these patients (p=0.014). (B) Kaplan-Meier curve of OS comparing patients who with bone metastasis, with a median OS of 14.7 months, who without bone metastasis, with a median OS of 17.1 months. No significant difference was identified between these patients (p=0.107). (C) Kaplan-Meier curve of PFS comparing patients who with axillary lymph nodes, with a median PFS of 4.8 months, who without axillary lymph nodes, with a median PFS of 3.9 months. No significant difference was identified between these patients (p=0.697). (D) Kaplan-Meier curve of OS comparing patients who with axillary lymph nodes, with a median OS of 15.3 months, who without axillary lymph nodes, with a median OS of 15.2 months. A statistically significant difference was identified between these patients (p=0.024). (E) Kaplan-Meier curve of PFS comparing patients who with brain metastasis, with a median PFS of 5.3 months, who without brain metastasis, with a median PFS of 4.7 months. No significant difference was identified between these patients (p=0.834). (F) Kaplan-Meier curve of OS comparing patients who with brain metastasis, with a median OS of 15.2 months, who without brain metastasis, with a median OS of 15.3 months. No significant difference was identified between these patients (p=0.673). (G) Kaplan-Meier curve of PFS comparing patients who with chest wall metastasis, with a median PFS of 5.1 months, who without chest wall metastasis, with a median PFS of 4.5 months. No significant difference was identified between these patients (p=0.769). (H) Kaplan-Meier curve of OS comparing patients who with chest wall metastasis, with a median OS of 15.7 months, who without chest wall metastasis, with a median OS of 15.2 months. No significant difference was identified between these patients (p=0.920). (I) Kaplan-Meier curve of PFS comparing patients who with liver metastasis, with a median PFS of 3.9 months, who without liver metastasis, with a median PFS of 5.1 months. No significant difference was identified between these patients (p=0.125). (J) Kaplan-Meier curve of OS comparing patients who with liver metastasis, with a median OS of 17.2 months, who without liver metastasis, with a median OS of 14.1 months. No significant difference was identified between these patients (p=0.869). (K) Kaplan-Meier curve of PFS comparing patients who with lung metastasis, with a median PFS of 5.0 months, who without lung metastasis, with a median PFS of 3.5 months. No significant difference was identified between these patients (p=0.456). (L) Kaplan-Meier curve of OS comparing patients who with lung metastasis, with a median OS of 16.6 months, who without lung metastasis, with a median OS of 14.1 months. No significant difference was identified between these patients (p=0.156).

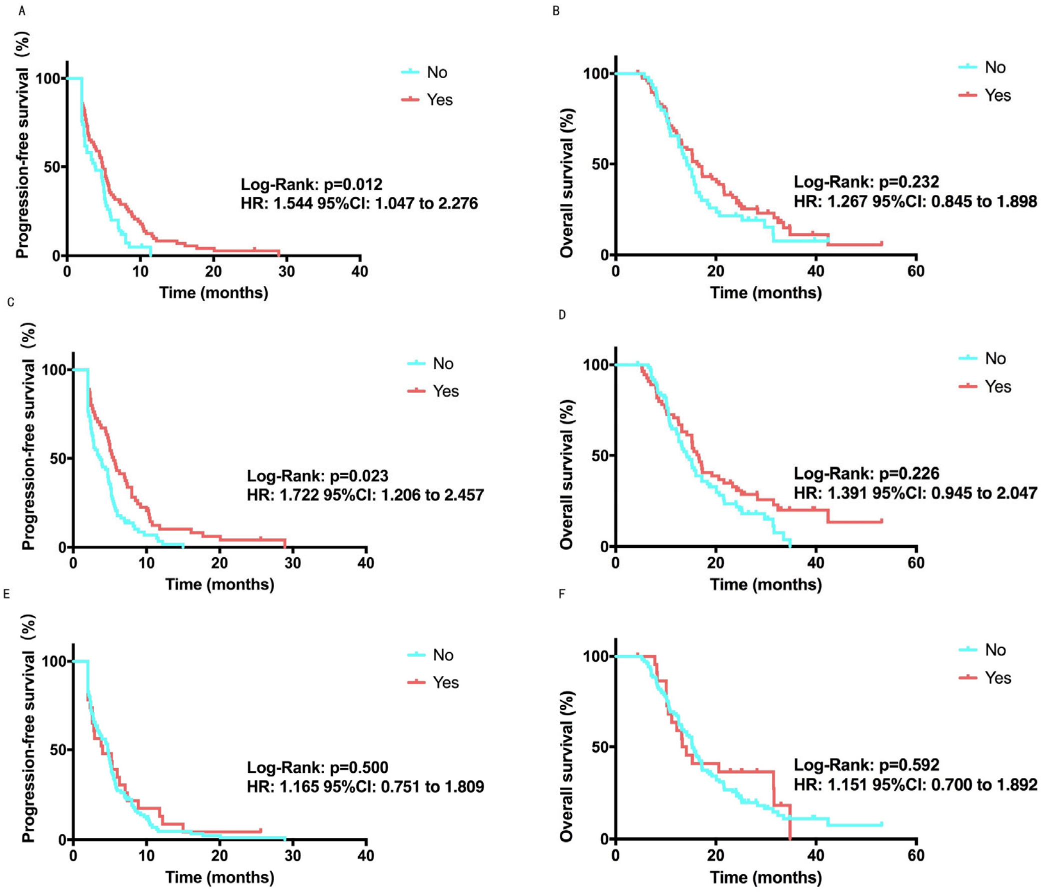

Figure S5. Association between PFS or OS and side effects after treatment. (A) Kaplan-Meier curve of PFS comparing patients who with hypertension, with a median PFS of 4.8 months, who without hypertension, with a median PFS of 3.8 months. A statistically significant difference was identified between these patients (p=0.012). (B) Kaplan-Meier curve of OS comparing patients who with hypertension, with a median OS of 16.2 months, who without hypertension, with a median OS of 14.2 months. No significant difference was identified between these patients (p=0.232). (C) Kaplan-Meier curve of PFS comparing patients who with hand-foot syndrome, with a median PFS of 5.3 months, who without hand-foot syndrome, with a median PFS of 3.9 months. A statistically significant difference was identified between these patients (p=0.023). (D) Kaplan-Meier curve of OS comparing patients who with hand-foot syndrome, with a median OS of 16.2 months, who without hand-foot syndrome, with a median OS of 14.7 months. No significant difference was identified between these patients (p=0.226). (E) Kaplan-Meier curve of PFS comparing patients who with proteinuria, with a median PFS of 4.0 months, who without proteinuria, with a median PFS of 4.7 months. No significant difference was identified between these patients (p=0.500). (F) Kaplan-Meier curve of OS comparing patients who with proteinuria, with a median OS of 13.7 months, who without proteinuria, with a median OS of 15.3 months. No significant difference was identified between these patients (p=0.592).

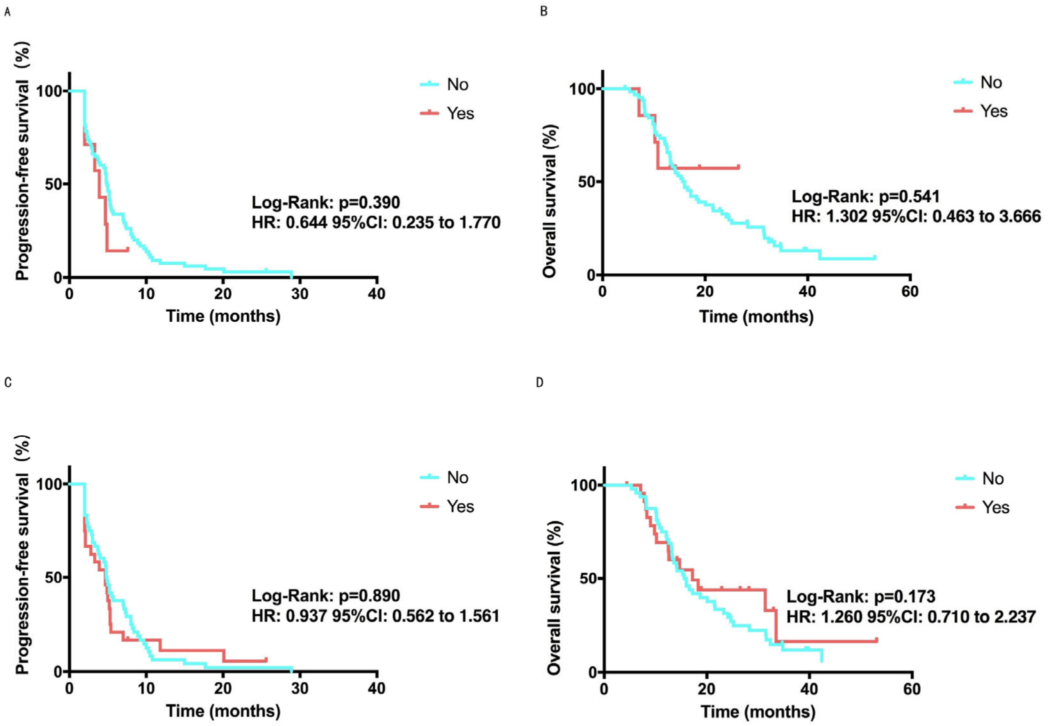

Figure S6. Kaplan-Meier curves of PFS and OS in pathology analysis. (A) Kaplan-Meier curve of PFS comparing patients who with micropapillary, with a median PFS of 3.9 months, and those who without micropapillary, with a median PFS of 4.8 months. No significant difference was identified between these patients (p=0.390). (B) Kaplan-Meier curve of OS comparing patients who with micropapillary, with a not reached median OS, and those who without micropapillary, with a median OS of 15.3 months. No significant difference was identified between these patients (p=0.514). (C) Kaplan-Meier curve of PFS comparing patients who with vascular cancer embolus, with a median PFS of 4.7 months, and those who without vascular cancer embolus, with a median PFS of 4.7 months. No significant difference was identified between these patients (p=0.890). (D) Kaplan-Meier curve of OS comparing patients who with vascular cancer embolus, with a median OS of 17.2 months, and those who without vascular cancer embolus, with a median OS of 15.2 months. No significant difference was identified between these patients (p=0.173).
